# Fitter Mitochondria Are Associated With Radioresistance in Human Head and Neck SQD9 Cancer Cells

**DOI:** 10.3389/fphar.2020.00263

**Published:** 2020-03-13

**Authors:** Debora Grasso, Hyllana C. D. Medeiros, Luca X. Zampieri, Vanesa Bol, Pierre Danhier, Marike W. van Gisbergen, Caroline Bouzin, Davide Brusa, Vincent Grégoire, Hubert Smeets, Alphons P. M. Stassen, Ludwig J. Dubois, Philippe Lambin, Marie Dutreix, Pierre Sonveaux

**Affiliations:** ^1^Pole of Pharmacology, Institut de Recherche Expérimentale et Clinique (IREC), Université catholique de Louvain (UCLouvain), Brussels, Belgium; ^2^Federal University of ABC – Universidade Federal do ABC (UFABC), São Paulo, Brazil; ^3^Pole of Molecular Imaging, Radiotherapy and Oncology (MIRO), Institut de Recherche Expérimentale et Clinique (IREC), UCLouvain, Brussels, Belgium; ^4^Nuclear and Electron Spin Technologies (NEST) Platform, Louvain Drug Research Institute (LDRI), UCLouvain, Brussels, Belgium; ^5^The M-Lab, Department of Precision Medicine, GROW - School for Oncology and Developmental Biology, Maastricht University, Maastricht, Netherlands; ^6^IREC Imaging Platform (2IP), Institut de Recherche Expérimentale et Clinique (IREC), UCLouvain, Brussels, Belgium; ^7^IREC Flow Cytometry and Cell Sorting Platform, Institut de Recherche Expérimentale et Clinique (IREC), UCLouvain, Brussels, Belgium; ^8^Centre Léon Bérard, Lyon, France; ^9^Department of Genetics and Cell Biology - GROW-School for Oncology and Developmental Biology, Maastricht University, Maastricht, Netherlands; ^10^Institut Curie, PSL Research University, CNRS UMR 3347, INSERM U1021, Paris-Sud University, Orsay, France

**Keywords:** Head and neck cancer, radiotherapy, radioresistance mechanisms, cancer metabolism, mitochondria, oxidative phosphorylation (OXPHOS)

## Abstract

The clinical management of head and neck squamous cell carcinoma (HNSCC) commonly involves chemoradiotherapy, but recurrences often occur that are associated with radioresistance. Using human SQD9 laryngeal squamous cell carcinoma cancer cells as a model, we aimed to identify metabolic changes associated with acquired radioresistance. In a top-down approach, matched radiosensitive and radioresistant SQD9 cells were generated and metabolically compared, focusing on glycolysis, oxidative phosphorylation (OXPHOS) and ROS production. The cell cycle, clonogenicity, tumor growth in mice and DNA damage-repair were assessed. Mitochondrial DNA (mtDNA) was sequenced. In a bottom-up approach, matched glycolytic and oxidative SQD9 cells were generated using FACS-sorting, and tested for their radiosensitivity/radioresistance. We found that acquired radioresistance is associated with a shift from a glycolytic to a more oxidative metabolism in SQD9 cells. The opposite was also true, as the most oxidative fraction isolated from SQD9 wild-type cells was also more radioresistant than the most glycolytic fraction. However, neither reduced hexokinase expression nor OXPHOS were directly responsible for the radioresistant phenotype. Radiosensitive and radioresistant cells had similar proliferation rates and were equally efficient for ATP production. They were equally sensitive to redox stress and had similar DNA damage repair, but radioresistant cells had an increased number of mitochondria and a higher mtDNA content. Thus, an oxidative switch is associated with but is not responsible for acquired radioresistance in human SQD9 cells. In radioresistant cells, more abundant and fitter mitochondria could help to preserve mitochondrial functions upon irradiation.

## Introduction

Head and neck squamous cell carcinoma (HNSCC) is the 6th most frequent and the 8th deadliest cancer type worldwide, accounting for ∼5% of all malignancies (Ferlay et al., 2015). Common treatments include surgery, radiotherapy and chemoradiotherapy (Adelstein et al., 2017). From 2012 to 2025, estimations predict an increase of ∼12% of HNSCC patients who will be treated with radiotherapy in Europe (Borras et al., 2016). However, despite recent technological advances, the success rate of radiotherapy in HNSCC is still limited, which is mainly due to radioresistance and unacceptable side effects. To improve therapy, one therefore needs to first identify the causes of HNSCC radioresistance.

Anticancer effects of X- and γ-rays are mostly indirect, with water radiolysis generating reactive oxygen species (ROS) that cause secondary ionizations. DNA is a major target. When reacting with ROS, in particular with the hydroxyl radical, a DNA radical is formed and water is produced. However, this reaction is reversible and damage can be promptly repaired unless it is stabilized, typically through a reaction with O_2_ that forms DNA peroxides (Jordan and Sonveaux, 2012). When cancer cells try to replicate, DNA lesions evolve to single strand breaks (SSBs) and double strand breaks (DSBs) that, if abundant, are ultimately cytotoxic. Oxygen availability is thus a critical parameter for cancer radiotherapy, and many radiosensitizing strategies have been proposed that improve tumor oxygenation (Jordan and Sonveaux, 2012; Danhier et al., 2013). The oxygen enhancement effect links radiotherapy efficacy to cancer metabolism, as oxidative cancer cells in tumors *in vivo* and in closed systems *in vitro* promote hypoxia, hence radioresistance, whereas glycolytic cancer cells spare oxygen that can be used to stabilize DNA damage (Danhier et al., 2013).

While hypoxia causes microenvironmental radioresistance, there is sensibly less yet increasing information about metabolic influences on intrinsic radiosensitivity that would be independent of hypoxia. In a recent review, Cruz-Gregorio et al. (2019) highlighted that reprogramming energy metabolism is critical for the induction of radioresistance in head and neck cancer. For example, accelerating the rate of the pentose phosphate pathway (PPP) in Warburg-phenotype Herpes virus (HPV)-negative HNSCC cells can increase the production of NADPH that fuels antioxidant enzymes (Williams et al., 2014; Chen et al., 2018; Cruz-Gregorio et al., 2018). Some radioresistant HNSCC cancer cells can also overexpress glucose transporters (GLUTs) or glycolytic enzymes to promote glucose metabolism instead of glutamine metabolism, which is associated to fast energy production and biosynthesis for cell survival and repair (Yan et al., 2013; Mims et al., 2015; Jung et al., 2017). Mitochondria can further modulate radiosensitivity by fine-tuning the activity of superoxide dimutases (SODs) and ROS production (Qu et al., 2010; Holley et al., 2014; Li et al., 2017). Lipid metabolism (Mims et al., 2015) and autophagy (Moergel et al., 2010; Kuwahara et al., 2011) have also been proposed as contributors to radioresistance.

Using human HNSCC cells, Bansal et al. (2014) and Mims et al. (2015) generated a radioresistant clone by irradiating SCC-61 tongue squamous cell carcinoma cells with 8 × 2 Gy, followed by clonal selection. They reported that, compared to wild-type cells, the radioresistant SCC-61 clone had increased glucose uptake fueling glycolysis and the PPP, enhanced lipogenesis and a decreased OXPHOS rate (Mims et al., 2015). It also had improved redox defenses (Bansal et al., 2014). However, because of the selection protocol, it is difficult to estimate whether these metabolic differences resulted from clonal selection or from acquired radioresistance. To sort this out, we generated a new model of radiosensitive and radioresistant human SQD9 laryngeal squamous cell carcinoma cancer cells that were not cloned. We also isolated glycolytic and oxidative SQD9 cells from the bulk wild-type population. Producing this double model was not possible with other HNSCC cell lines. Paired cell lines were metabolically compared and were tested for intrinsic radiosensitivity/radioresistance in the presence of oxygen. For further characterization, we focused on mitochondria that control apoptosis and ATP and ROS production, and that contain their own DNA that could be a target of radiotherapy.

## Materials and Methods

### Cells and Cell Culture

Wild-type HPV/p16-negative SCC9, SCC61 and Cal27 cells were from ATCC (Manassas, United States). Wild-type HPV/p16-negative SQD9 cells (SQD9-wt) were a kind gift of AC Begg (The Netherlands Cancer Institute). All cells were routinely cultured in Dulbecco’s modified Eagle’s medium (DMEM #61965-026; Gibco Life technologies, Erembodegem, Belgium) containing 4.5 g/L of glucose, 2 mM of glutamax and supplemented with 10% fetal bovine serum (FBS). SQD9 radioresistant cells (SQD9-res) were obtained by a chronic exposure (2 weeks) to low irradiation doses (2 Gy/day) delivered by a ^137^Cs γ-irradiator (IBL637, ORIS, France) at a dose rate of 0.80 Gy/min. SCC9, SCC61 and Cal27 cells were treated the same way. Cells were cultured for 2 additional weeks before experiments. SQD9 cells with high glucose uptake (SQD9-HGU) and SQD9 cells with low glucose uptake (SQD9-LGU) were obtained by treating SQD9-wt cells for 2 h with 50 μM of 2-(*N*-(7-nitrobenz-2-oxa-1,3-diazol-4-yl)amino)-2-deoxyglucose (2-NBDG; Thermo Fisher Scientific, Merelbeke, Belgium), followed by FACS-sorting of the 2% cells that were the most positive (SQD9-HGU) and the 2% cells that were the less positive (SQD9-LGU) for 2-NBDG uptake on a FACSAria III (BD Biosciences, Erembodegem, Belgium). SCC9, SCC61 and Cal27 cells were sorted the same way. The quantification of 2-NBDG uptake by SQD9-wt and SQD9-res cells used the same protocol, but without cell sorting. Cell number was determined on a SpectraMax miniMax 300 imaging cytometer (Molecular Devices, Wokingham, United Kingdom).

### Cell Authentication

Cell lines were tested every 6 months with a short tandem repeat (STR) test. DNA was isolated with a QIAmp DNA kit (Qiagen, Antwerp, Belgium) following manufacturer’s instructions and amplified by PCR using the PowerPlex 16 System Promega amplification kit (Leiden, The Netherlands). Fifteen markers (D3S1358, THO1, D21S11, D18S51, Penta E, D5S818, D13S317, D7S820, D16S539, CSF1PO, Penta D, Vwa, D8S1179, TPOX, FGA and amelogenin) were used to obtain the genetic profile (Pole of Genetic Expertise, UCLouvain, Belgium).

### Cell Irradiation and Clonogenic Assays

Exponentially growing cancer cells (50–10,000 cells per well) were plated in 6-well plates. Where indicated, cells were treated for 1 h with 10 nM of antimycin A (Sigma-Aldrich, Overijse, Belgium). After adhesion, they were irradiated using a ^137^Cs γ-irradiator at a dose-rate of 0.80 Gy/min for total absorbed doses of 2, 4 or 8 Gy or treated for 48 h with H_2_O_2_ (Sigma-Aldrich), as indicated. For clonogenic assays, cells were incubated until cells in sham-irradiated control wells had formed colonies containing more than 50 cells. They were fixed and stained with 0.5% crystal violet in a 10% ethanol solution for 30–60 min, washed with water, air-dried and counted. The surviving fraction was expressed as the plating efficiency of treated/control cells. Results are displayed on a logarithmic scale.

### *In vivo* Experiments

All *in vivo* experiments were performed with approval of UCLouvain *Comité d’Ethique pour l’Expérimentation Animale* (approvals 2014/UCL/MD/014 and 2016/UCL/MD/018) according to national and European animal care regulations. Under anesthesia (80 mg/kg of ketamine and 8 mg/Kg of xylazine), SQD9-wt and SQD9-res cells were injected subcutaneously respectively in the left and right flanks of 7 weeks old male NMRI nude mice (Janvier, Le Genest-Saint-Isle, France), or *vice versa*, as a 2:1 v/v solution of 1,000,000 cells in HBSS:Matrigel (#356231, Corning). SQD9-HGU and SQD9-LGU cells were injected in the same manner to 7 weeks old male NMRI nude mice. Once tumors became palpable, their size was measured over time with an electronic caliper, and tumor volumes were calculated according to two different axes (D × d^2^ x π/6).

### Metabolic Assays

Glucose and lactate concentrations were measured using specific enzymatic assays on a CMA600 microdialysis analyzer (Kista, Sweden). For the determination of glucose uptake and lactate production rates, an equal number of cells were plated in 6-well plates with 2 ml of fresh medium. Seventy-two hours later, supernatants were collected and filtered on 10K tubes (VWR, Leuven, Belgium). Intracellular glucose and lactate concentrations were measured on the same system using a previously disclosed protocol (Sonveaux et al., 2008). Glucose consumption and lactate production were calculated and normalized by total protein content using the Bio-Rad protein assay (Temse, Belgium). Oxygen consumption rates (OCRs) were determined on a Seahorse XF96 bioenergetic analyzer using the XF cell mito stress kit (Agilent Technologies, Diegem, Belgium) according to manufacturer’s recommendations. Twenty thousand cells per well were plated on XF96 culture plates 24 h before experiments in complete DMEM containing 10% FBS. On the day of analysis, culture medium was replaced by DMEM (#D5030; Sigma-Aldrich) containing 1.85 g/L of NaCl, 3 mg/L of phenol red, 10 mM of glucose and 2 mM of glutamine, pH 7.4. Cells were incubated for 1 h in a CO_2_-free incubator before analysis. In the Seahorse analyzer, oximetry was repeatedly performed in closed wells after the sequential addition of the components of the XF cell mito stress kit: oligomycin to inhibit ATP-synthase, ionophore carbonyl cyanide-4-(trifluoromethoxy)phenylhydrazone (FCCP) to disrupt the mitochondrial potential, and rotenone together with antimycin A to simultaneously inhibit Complexes I and III of the mitochondrial electron transport chain (ETC). Oximetry before the addition of any agent provided the basal respiration rate of the cells, ATP-linked reparation was determined after the addition of 1 μM of oligomycin, the maximal respiration rate of the cells after the addition of 1 μM of FCCP, and non-mitochondrial oxygen consumption after the addition of 0.5 μM of rotenone together with 0.5 μM of antimycin A. All data were normalized to cell numbers measured right before oximetry using a SpectraMax miniMax 300 imaging cytometer. Cellular ATP levels were measured using the CellTiter-Glo Luminescent Viability assay (Promega) on a Glomax 96 microplate luminometer (Promega) following manufacturer’s instructions.

### Western Blotting

Western blotting was performed as previously described (Van Hee et al., 2015). Primary antibodies were: a rabbit polyclonal against GLUT1 (#652; Abcam, Cambridge, United Kingdom); rabbit monoclonals against GLUT3 (#191071; Abcam), HK 1/2 (#2024/2867; Cell Signaling, Leiden, The Netherlands), PKM2 (#4053; Cell Signaling) p21 Waf1/Cip1 (12D1) (#2947; Cell Signaling), p53 (7F5) (#2527; Cell Signaling), ATR (#2790; Cell Signaling), phospo-Ser428-ATR (#2853; Cell Signaling), ATM (D2E2) (#2873; Cell Signaling), phospho-Ser1981-ATM (D6H9) (#5883; Cell Signaling); and mouse monoclonals against phospho-Ser15-p53 (#9286; Cell Signaling) and β-actin (#A5441; Sigma-Aldrich). Secondary antibodies were HRP-coupled goat anti-rabbit and anti-mouse (Jackson ImmunoResearch, Huissen, The Netherlands). Staining was revealed with an Amersham Imager 600 (GE Healthcare, Diegem, Belgium). Data were analyzed using the ImageJ software (NIH, Bethesda, United States).

### Gene Silencing

Silencing was performed by using a mix of siRNAs for HK1 (siRNAs ID #s6557 and #s6558; Life technologies) and for HK2 (siRNAs ID #s6560 and #s6561; Life technologies). Incubation was performed for 48 h in lipofectamine RNAiMAX (Invitrogen) and OPTI-MEM (Gibco). Cells were used directly after lipofection.

### mtDNA

mtDNA was sequenced at four different cell passages. Enrichment of the entire mtDNA genome was performed by a two fragments long-range PCR using the Phusion Hot Start DNA polymerase II kit (Thermo Fisher Scientific) and 100 ng of total genomic DNA, according to manufacturer’s guidelines. The two mtDNA fragments (8.2 kb PCR fragment I and 8.5 kb PCR fragment II) were purified using Agencourt AMPure XP beads (Beckman Coulter, Suarlée, Belgium). Bound PCR fragments were washed with 70% ethanol and eluted using Qiagen Buffer EB. Fragments I and II were pooled for every sample, and mtDNA concentrations were determined using the Qubit High Sensitivity kit (Thermo Fisher Scientific). Library preparation was performed using the Illumina Nextera XT kit (Illumina, Eindhoven, The Netherlands). Each sample had a unique combination of index adapters (sequences), enabling dual indexing. All samples were equimolarly pooled and sequenced in a single lane of 1 MiSeq flow-cell using the MiSeq Reagent Kit v3 (600-cycle) with 2 × 300 bp paired-end chemistry (Illumina). mtDNA sequencing was performed on an Illumina Miseq system. Data demultiplexing was performed by using Illumina CASAVA software (v.1.8.4.). The reads were aligned against the revised Cambridge reference sequence (rCRS; except the gap present at position 3,107) by the BWA software (v.0.5.9.) (Li and Durbin, 2009). For the identification of variants and small indels (insertions or deletions of bases), Python v2.6.6, Python package pysam v0.7.8 and SAMTools v0.1.19 softwares were used (Li and Durbin, 2009). Heteroplasmy levels were calculated by the read depth of the variant *versus* reference nucleotides on every nucleotide position. A cut-off of >2% was used to determine heteroplasmy levels, whereas a cut-off of >80% of heteroplasmy was considered as a homoplasmic variant.

mtDNA copy number was determined following the procedure detailed by Rooney et al. (2015). Briefly, total DNA was isolated with a QIAmp DNA kit (Qiagen) following manufacturer’s instructions. RT-qPCR was performed using TaqMan universal master mix II with UNG (Applied Biosystems, Merelbeke, Belgium) on a ViiA 7417 Real-Time instrument (Life Technologies). Nuclear DNA was quantified using as primers RNAseP VIC (2’-chloro-7’phenyl-1,4-dichloro-6-carboxy-fluorescein) labeled probe (#4401631; Thermo Fisher Scientific). mtDNA was quantified using as primers: forward: 5′-GTA CCC ACG TAA AGA CGT TAG G-3′; reverse: 3′-TAC TGC TAA ATC CAC CTT CG-5′; and as labeled probe 5′-CCC ATG AGG TGG CAA GAA AT-3′ FAM (5(6)-carboxyfluorescein). mtDNA content was normalized to nuclear DNA content, as previously done (Li and Durbin, 2009).

### ROS Measurements

Intracellular ROS were measured using carboxymethyl-2′,7′-dichlorofluorescein diacetate (CM-H_2_DCFDA, Sigma-Aldrich), as previously reported (Porporato et al., 2014).

### γH2AX and Cell Cycle Analyses

Cells were harvested using trypsin-EDTA, fixed with ethanol 70% and permeabilized with 0.1% Triton X-100. AlexaFluor488 anti-γ-H2AX antibody (#560445, BD Biosciences) was added in a 1:5 dilution and incubated at room temperature for 1 h in dark conditions. Propidium iodide (5 μg) was added to 10^6^ cells to identify cell cycle phases. Cell analyses were performed acquiring at least 20,000 events on a BD FACSCantoII flow cytometer using FACSDiva (BD Biosciences) and FlowJo (FlowJo, LLC) softwares.

### Alkaline Comet Assay

Single-cell gel electrophoresis was carried out using the Trevigen alkaline CometAssay Kit (#4250-050; Bio-Techne, Lille, France) following the procedure detailed by Quanz et al. (2009). DNA breaks were assessed immediately and at the indicated times after irradiation. DNA damage was quantified as the Comet tail moment, which represents the extent of DNA damage [both single strand breaks (SSBs) and double strand breaks (DSBs) in individual cells; Azqueta et al., 2014].

### Immunofluorescence and Analysis of Mitochondrial Morphology

Immunofluorescent assays were performed on cells cultured on glass coverslips. Cells were fixed in 4% formaldehyde, permeabilized with 0.1% Triton X-100 in PBS containing 0.5% Tween20, and blocked with 5% BSA. Primary antibody was a rabbit polyclonal against mitochondrial import receptor subunit TOM20 (#PA5-52843, 1/200; Invitrogen, Merelbeke, Belgium) that had been previously validated by others using shRNAs (Cheng et al., 2009). Secondary antibodies were goat anti-rabbit Alexa Fluor 488 (#A-11,034, 1/1,000; Invitrogen). For the staining of F-actin, we used phalloidin-650 (1/250; Thermo Fisher). Nuclei were stained with 4′,6-diamidino-2-phenylindole dihydrochloride (DAPI) (1 μg/μl; Sigma-Aldrich). Mitochondrial network analysis was performed using the MiNa toolset on ImageJ following a previously described methodology (Valente et al., 2017). Cells were counted in 28 different random fields. Images of mitochondrial networks were captured by structured illumination fluorescence microscopy using an ApoTome-equipped AxioImager.z1 microscope (Zeiss, Zaventem, Belgium).

### Statistics

All data are expressed as means ± SEM. Error bars are sometimes smaller than symbols. *n* refers to the total number of replicates per group. Each experiment was repeated at least twice independently, except cell cycle measurements, γ-H2AX measurements and the Comet assay where > 250 cells per group were analyzed. Data were analyzed using GraphPad Prism 7.0 (GraphPad Software, La Jolla, CA, United States). Survival curve fitting was performed using the “Fit” function from Matlab. Student’s *t*-test, one-way ANOVA with Dunnett’s *post hoc* test and two-way ANOVA with Sidak’s multiple comparison test were used where appropriate. *P* < 0.05 was considered to be statistically significant.

## Results

### Model Generation

**FIGURE 1:**
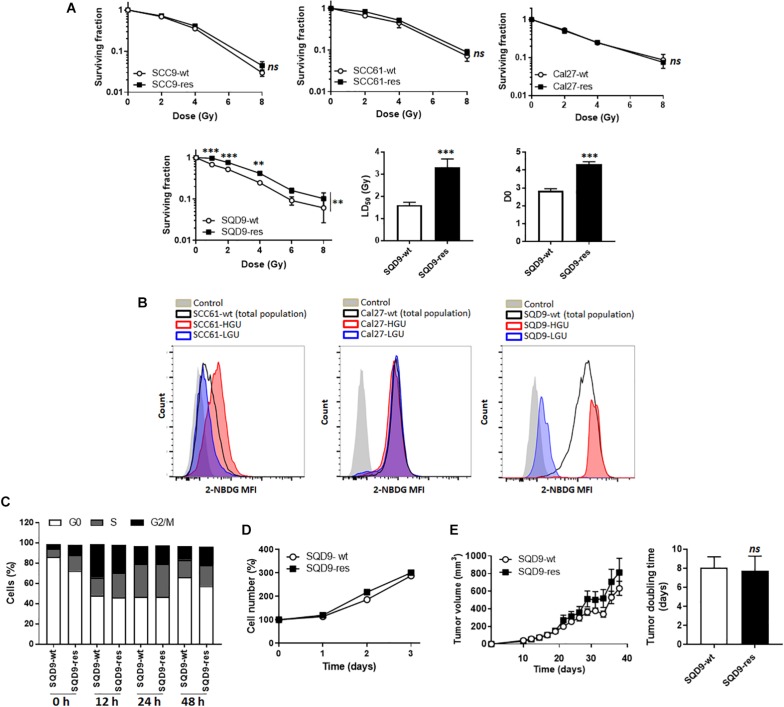
Model selection. **(A)** From left to right: surviving fractions of SCC9-wt *versus* SCC9-res (*n* = 3), SCC61-wt *versus* SCC61-res (*n* = 3), Cal27-wt *versus* Cal27-res (*n* = 2) and SQD9-wt *versus* SQD9-res cells (*n* = 6). For SQD9-wt and SQD9-res cells, two additional graphs show the lethal dose 50 (LD_50_) and the D_0_ dose (*n* = 6). **(B)** Wild-type cells were FACS-sorted to isolate the 2% subpopulation of cells with the highest uptake of glucose analog 2-NBDG (HGU cells) and the 2% subpopulation with the lowest 2-NBDG uptake (LGU cells). The representative images were acquired after 2 weeks of culture following FACS sorting. They show counts in function of 2-NBDG mean fluorescence intensity for SCC61, Cal27 and SQD9 cells. Control corresponds to untreated cells. **(C)** Cell cycle analyzed over time (h) after a 8 Gy irradiation (*n* = 2). **(D)** Cell number measured over time in non-irradiated cells (*n* = 8). **(E)** SQD9-wt and SQD9-res *in vivo* tumor growth is shown on the left graph and tumor doubling times on the right graph. Tumors were not irradiated (*n* = 11–12). ^∗∗^*P* < 0.01, ^∗∗∗^*P* < 0.005, *ns P* > 0.05 by two-way ANOVA followed by a Sidak’s multiple comparison test **(A)** or Student’s *t-*test (**A** LD_50_ and D_0_; **E** *right*).

In this study, we decided to use a top-down and a bottom-up approach to test metabolic influences on radiosensitivity in HNSCC cells. For the top-down approach, we tried to generate radioresistant HNSCC cells by treating SCC9, SCC61, Cal27 and SQD9-wt cells with daily 2 Gy doses of γ-rays for 2 weeks. Cell identity was confirmed using STR DNA profiling at the end of treatment. Among these 4 cell lines, only SQD9-wt cells generated radioresistant cells, as validated *in vitro* with clonogenic assays that revealed that SQD9-res had a higher surviving fraction than SQD9-wt cells after irradiation with increasing doses of γ-rays under normoxia (Figure 1A).

For the bottom-up approach, we tried to isolate subpopulations of highly glycolytic and highly oxidative cancer cells using FACS-sorting for glucose uptake with the fluorescent glucose analog 2-NBDG. The 2% fraction of cells that were the most avid (HGU; high glucose uptake) and the 2% of cells that were the least avid (LGU; low glucose uptake) for 2-NBDG were isolated. Two weeks after sorting, metabolically different subpopulations persisted only in the SQD9-LGU/SQD9-HGU model, but not in SCC61 and Cal27 series (Figure 1B). We therefore decided to further characterize the SQD9-wt/SQD9-res and SQD9-LGU/SQD9-HGU pairs of matched HNSCC cells.

When comparing SQD9-wt to SQD9-res cells, the most significant differences were seen between 1 and 4 Gy, and the lethal dose necessary to kill 50% of the cells (LD_50_) was ∼2-fold higher for SQD9-res compared to SQD9-wt cells (Figure 1A). Surviving curves fitted with a shouldered model with zero initial slope, *i.e.*, almost straight survival curves at low doses followed by shouldered curves, where some cells die after 1 hit following the single target single-hit model and others after several hits following the multitarget model. This indicated that both SQD9-wt and SQD9-res cells constituted a heterogeneous cell population that significantly differed for the D_0_ dose (Figure 1A). *In vitro* cell cycling after irradiation (Figure 1C), cell proliferation without treatment (Figure 1D) and *in vivo* tumor growth without treatment (Figure 1E) were similar for SQD9-wt and SQD9-res cells.

### Acquired Radioresistance Is Associated With a Switch From a Glycolytic to a More Oxidative Metabolism in SQD9 Cancer Cells

**FIGURE 2:**
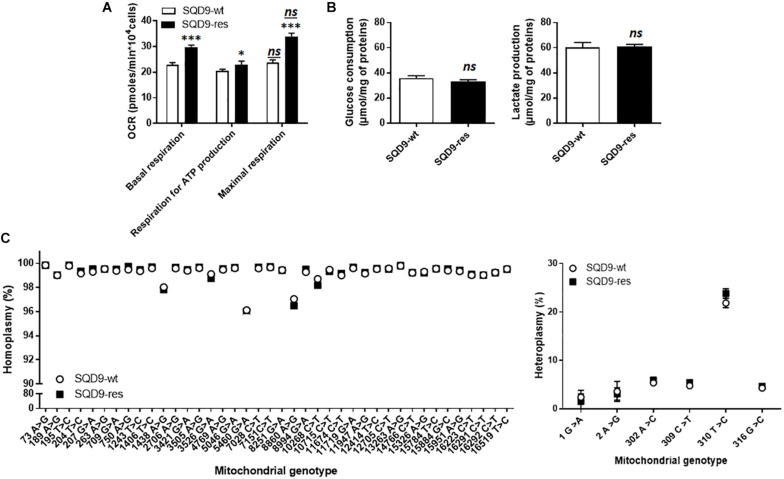
Acquired radioresistance of SQD9 cells correlates with increased mitochondrial respiration. **(A)** OCRs corresponding to basal respiration, maximal respiration and the oxidative contribution to ATP production (*n* = 24). **(B)** Glucose consumption (*left*) and lactate production (*right*) 72 h after cell seeding (*n* = 8–9). **(C)** mtDNA was sequenced. The left graph shows the comparative distribution of homoplasmic variants and the right graph heteroplasmic variants (*n* = 4). ^∗^*P* < 0.05, ^∗∗∗^*P* < 0.005, *ns P* > 0.05 for the same parameter between SQD9-wt and SQD9-res; *ns* *P* > 0.05 *versus* basal respiration of the same cell line; by Student’s *t-*test **(A–C)**.

Metabolic comparison revealed that SQD9-res cells had a more oxidative phenotype than SQD9-wt, as they had a higher OCR associated to basal and maximal respiration and an increased dependency on mitochondrial metabolism to generate ATP (Figure 2A). Of note, SQD9-wt and SQD9-res cells were respiring at their maximal capacity. No difference was observed in the rates of glucose consumption and lactate production (Figure 2B). Thus, acquired radioresistance was associated with increased OXPHOS yet unaltered glycolysis coupled to lactic fermentation.

Because irradiation is well known to damage DNA and because the metabolic changes that we observed affected mitochondria, we investigated the possibility of mtDNA defects in our model. Compared to the revised Cambridge reference sequence, all observed homoplasmic mtDNA variants were present in SQD9-wt and in SQD9-res cells (Figure 2C, *left*), but no unique homoplasmic mtDNA variant was detected. No differences were found for heteroplasmic mtDNA either (Figure 2C, *right*). Thus, a chronic irradiation protocol did not select for mtDNA abnormalities in SQD9 cells.

### Oxidative SQD9 Are More Radioresistant Than Glycolytic SQD9 Cancer Cells

**FIGURE 3:**
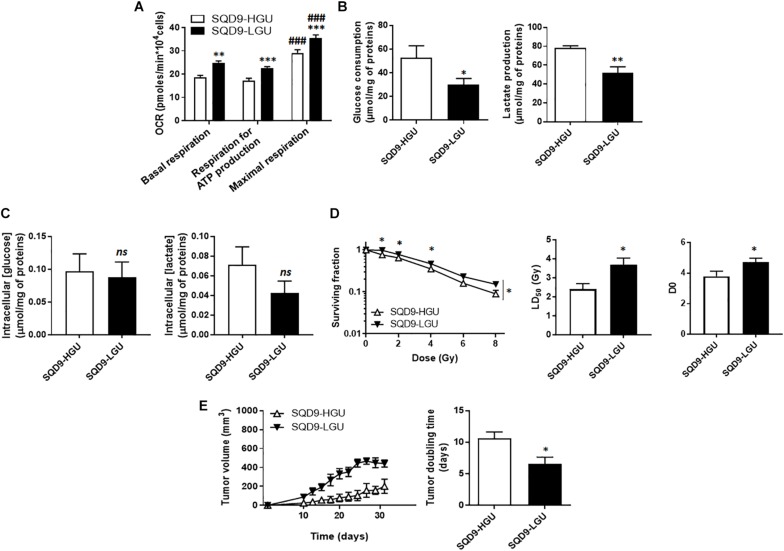
Oxidative SQD9 are more radioresistant than glycolytic ones. **(A)** OCRs corresponding to basal respiration, maximal respiration and the oxidative contribution to ATP production (*n* = 16). **(B)** Glucose consumption (*left*) and lactate production (*right*) 72 h after cell seeding (*n* = 5–6). **(C)** Intracellular glucose and lactate concentrations normalized by total protein expression (*n* = 4–10). **(D)** Clonogenicity of cells irradiated at increasing doses of Ɣ-rays. The left graph shows surviving fractions, the middle graph LD_50_ and the right graph the D_0_ dose (*n* = 9). **(E)** *In vivo* SQD9-HGU and SQD9-LGU tumor growth is shown on the left graph, and tumor doubling times on the right graph. Tumors were not irradiated (*n* = 5–6). ^∗^*P* < 0.05, ^∗∗^*P* < 0.01, ^∗∗∗^*P* < 0.005; **ns* P* > 0.05 for the same parameter between SQD9-HGU and SQD9-LGU; ^###^*P* < 0.005 *versus* basal respiration of the same cell line; by Student’s *t-*test (**A–C**,**D** *middle*, **D** *right*, **E** *right*); or two-way ANOVA followed by a Sidak’s multiple comparison test (**D** *left*).

For the SQD9-LGU/SQD9-HGU pair of matched cells, metabolic characterization revealed that SQD9-LGU cells had increased basal and maximal OCR and a higher dependency on OXPHOS to generate ATP (Figure 3A). They consumed less glucose and produced less lactate than SQD9-HGU cells (Figure 3B). In both cases, cells did not use their maximal respiration capacity. We measured similar intracellular glucose and lactate levels in SQD9-HGU and SQD9-LGU cells (Figure 3C), thus indicating that glucose was efficiently processed in both cell lines. We concluded that SQD9-LGU cells were a more oxidative subpopulation compared to SQD9-HGU cells that were more glycolytic.

From a radiosensitivity standpoint, clonogenic assays revealed that oxidative SQD9-LGU were more radioresistant than glycolytic SQD9-HGU cells (Figure 3D). The most significant differences were seen between 1 and 4 Gy, and the lethal dose necessary to kill 50% of the cells (LD_50_) was ∼1.5-fold higher for SQD9-LGU compared to SQD9-HGU cells. The D_0_ dose was also significantly increased for SQD9-LGU cells (Figure 3D). Curve fitting followed the same model as in Figure 1A, indicating that SQD9-LGU and SQD9-HGU still constituted heterogeneous cell populations. In the absence of irradiation, SQD9-LGU cells also generated more aggressive tumors *in vivo* (Figure 3E), with unaltered grafting efficiency.

**FIGURE 4:**
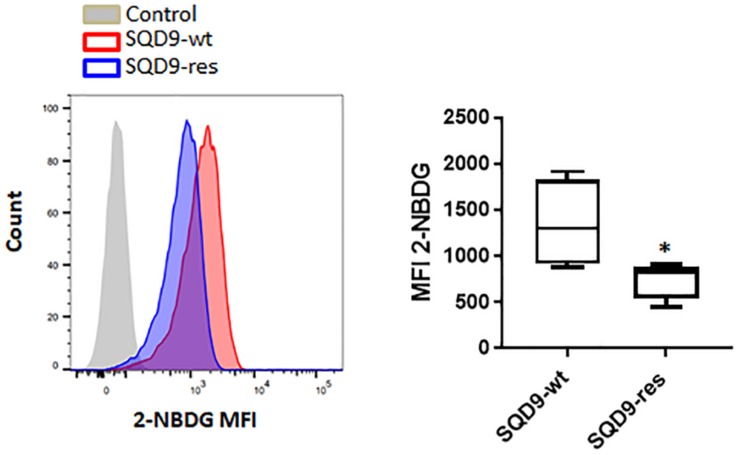
SQD9-res cells take up and sequester less 2-NBDG than SQD9-wt cells. SQD9-wt and SQD9-res cells were treated for 2 h with 2-NBDG, and analyzed by FACS. The left representative image shows cell count in function of 2-NBDG mean fluorescence intensity (MFI) and the right graph shows the quantified MFI (*n* = 3). ^∗^*P* < 0.05; by Student’s *t-*test.

At this stage, our top-down (Figures 1, 2) and bottom-up (Figure 3) approaches indicated that radioresistance is associated with an oxidative metabolism and that an oxidative metabolism is associated with radioresistance in our model. That oxidative SQD9-res behaved like oxidative SQD-LGU cells was further illustrated by the fact that SQD9-res cells had a low 2-NBDG uptake rate compared to SQD9-wt cells (Figure 4). Conversely, SQD9-wt and SQD9-HGU cells both efficiently took up and sequestered 2-NBDG (Figures 1B, 4).

### The Oxidative Metabolism of Radioresistant SQD9 Cells Is Associated With a Decrease in the Expression of Hexokinases

**FIGURE 5:**
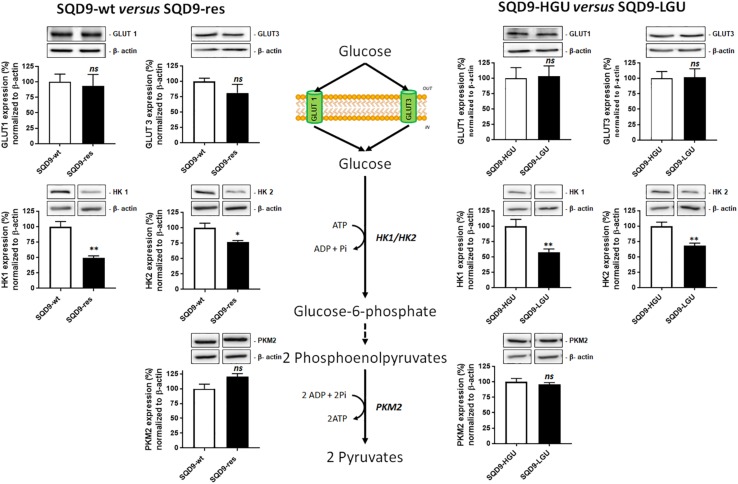
Oxidative SQD9-res and SQD9-LGU cells are characterized by a glycolytic bottleneck at the level of hexokinases. The figure depicts glycolysis where glucose transporters GLUT1 and GLUT3 facilitate glucose uptake by cancer cells, hexokinases 1 and 2 (HK1 and HK2) catalyze glucose phosphorylation to yield glucose-6-phosphate, and pyruvate kinase M2 (PKM2) the conversion of phosphoenolpyruvate to pyruvate. The expression of the 2 transporters and the 3 enzymes was analyzed using western blotting in SQD9-wt *versus* SQD9-res (on the *left*) and in SQD9-HGU *versus* SQD9-LGU (on the *right*). On top of each quantification graph, representative pictures were each time produced on a same membrane (*n* = 4–6). ^∗^*P* < 0.05, ^∗∗^*P* < 0.01; **ns* P* > 0.05; by Student’s *t-*test.

We next aimed to identify the origin of the differences in 2-NBDG uptake between our model cell lines. 2-NBDG is an analog of 2-deoxyglucose (2DG), which is imported by GLUTs, phosphorylated by hexokinases (HKs), and, at the difference of glucose, accumulates in cells because it cannot be further metabolized (Millon et al., 2011). We therefore focused on the upstream part of glycolysis, comparing more oxidative (SQD9-res and SQD9-LGU) to more glycolytic (SQD9-wt and SQD9-HGU) cells. Western blot analyses revealed that both SQD9-res and SQD9-LGU cells had decreased expression of HK1 and HK2 compared to SQD9-wt and SQD9-HGU cells, respectively (Figure 5). We detected no changes in the expression of GLUT1, GLUT3 and pyruvate kinase M2 (PKM2) (Figure 5). Because HKs are key glycolytic enzymes that are responsible for the phosphorylation/sequestration of 2-NBDG (Millon et al., 2011), we concluded that their reduced expression accounted for the low 2-NBDG uptake of SQD9-res and SQD9-LGU cells. This set of data further illustrated the similarity between the two radioresistant cell lines.

### Silencing Hexokinases Does Not Selectively Kill Radioresistant SQD9 Cells

**FIGURE 6:**
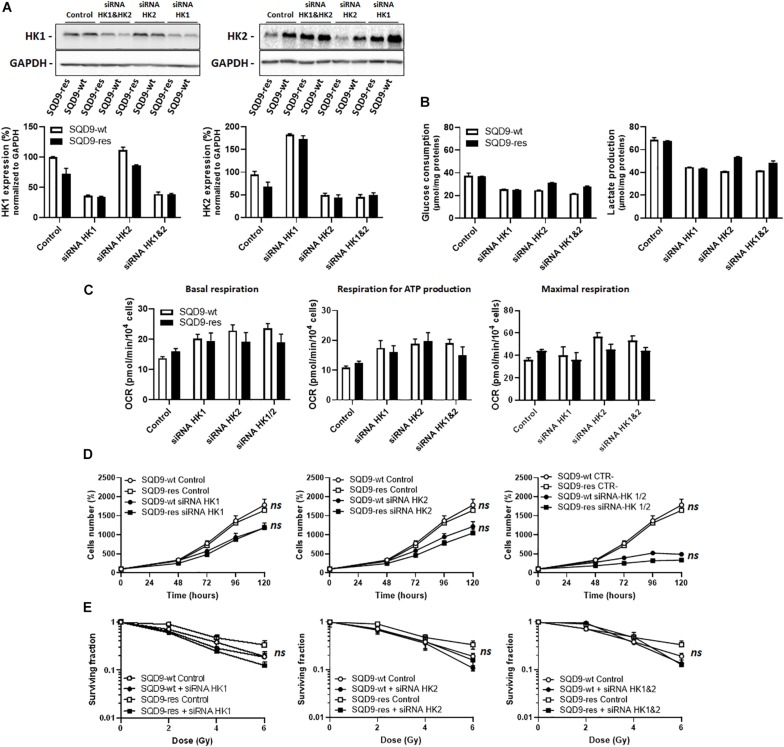
Silencing hexokinases in SQD9-wt and SQD9-res cells. (**A–E**) SQD9-wt and SQD-9-res cells were lipofected with a cocktail of siRNAs targeting either HK1 or HK2 or both together. **(A)** Representative western blots show the expression of HK1, HK2 and GAPDH, which is quantified in the graphs (*n* = 2). **(B)** Glucose consumption (*left*) and lactate production (*right*) 72 h after cell seeding (*n* = 2). **(C)** OCRs corresponding to basal respiration, maximal respiration and the oxidative contribution to ATP production (*n* = 3–5). **(D)** Cell number measured over time (*n* = 4). **(E)** Clonogenic assay after irradiation at the indicated doses, where the graphs show surviving fractions (*n* = 4). *ns P* > 0.05 two-way ANOVA followed by a Sidak’s multiple comparison test **(D,E)**.

Based on the above observations, we next envisioned that targeting HK1 and/or HK2 could potentially selectively kill radioresistant SQD9-res and SQD-9-LGU cells. We first focused on the SQD9-wt/SQD9-res matched pair of cells. Cocktails of siRNAs silencing either *HK1*, *HK2* or both were validated using western blotting, where the expression of more than half of the targeted proteins was lost (Figure 6A). Silencing *HK1* decreased glucose consumption and lactate release by SQD9-wt and SQD9-res cells, but there was no selective effect on radioresistant cells (Figure 6B). Cell respiration increased, but, here again, there was no difference between the cell lines (Figure 6C). Silencing *HK1* further decreased cell expansion *in vitro* (Figure 6D), but had no differential effect on the radiosensitivity of SQD9-wt *versus* SQD9-res cells (Figure 6E). Silencing *HK2* had similar effects than silencing *HK1* in all assays (Figures 6B–E). Silencing *HK1* and *HK2* together further repressed cell expansion *in vitro* (Figure 6D, *right*), but did not preferentially radiosensitize SQD9-res compared to SQD9-wt cells (Figure 6E, *right*). It was not possible to achieve better silencing of HKs because of the long half-life of the proteins and repression of cell proliferation by the silencing.

**FIGURE 7:**
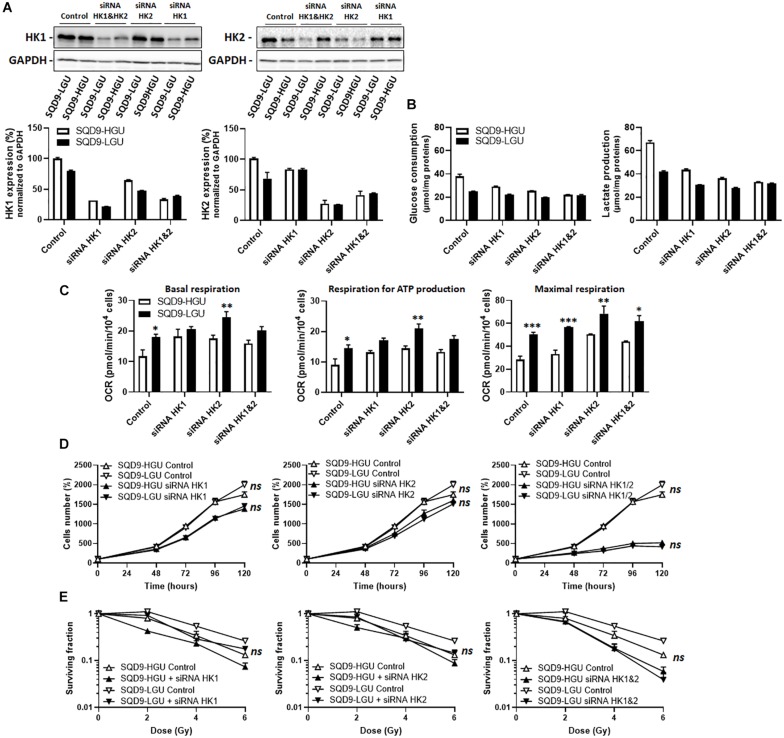
Silencing hexokinases in SQD9-HGU and SQD9-LGU cells. **(A–E)** SQD9-HGU and SQD-9-LGU cells were lipofected with a cocktail of siRNAs targeting either HK1 or HK2 or both together. **(A)** Representative western blots show the expression of HK1, HK2 and GAPDH, which is quantified in the graphs (*n* = 2). **(B)** Glucose consumption (*left*) and lactate production (*right*) 72 h after cell seeding (*n* = 2). (**C**) OCRs corresponding to basal respiration, maximal respiration and the oxidative contribution to ATP production (*n* = 3–5). **(D)** Cell number measured over time (*n* = 4). **(E)** Clonogenic assay after irradiation at the indicated doses, where the graphs show surviving fractions (*n* = 4). *P < 0.05, **P < 0.01, ***P < 0.005, *ns P* > 0.05 by student’s *t*-test **(C)** or by two-way ANOVA followed by a Sidak’s multiple comparison test **(D,E)**.

These experiments were repeated targeting HKs in SQD9-HGU and SQD9-LGU. siRNA-mediated repression of HK protein expression was achieved to the same degree as in SQD9-wt and SQD9-res cells (Figure 7A). Silencing *HK1* or *HK2* repressed glucose consumption and lactate release in both SQD9-HGU and SQD9-LGU cells, yet SQD9-HGU kept a higher glycolytic rate than SQD9-LGU cells (Figure 7B). This difference was lost when silencing both HKs together. Silencing *HK1*, *HK2* or both also increased the respiration rate of both cell lines, yet SQD9-LGU generally still kept a higher OCR than SQD9-HGU (Figure 7C). Silencing *HK1* or *HK2* decreased cell expansion *in vitro* to the same extent (Figure 7D), but had no differential effect on the radiosensitivity of SQD9-LGU *versus* SQD9-HGU cells (Figure 7E). Silencing *HK1* and *HK2* together further repressed cell expansion *in vitro* (Figure 7D, *right*), but did not preferentially radiosensitize SQD9-LGU compared to SQD9-HGU cells (Figure 7E, *right*).

Together, these experiments indicated that the lower expression of HKs in radioresistant SQD9 cells could not be exploited for selective radiosensitization using genetic approaches.

### OXPHOS and Redox Defenses Do Not Account for Acquired Radioresistance in SQD9 Cancer Cells

**FIGURE 8:**
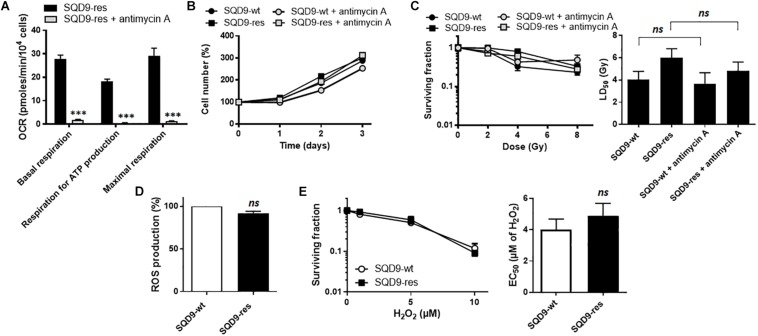
OXPHOS and redox changes are not responsible for acquired radioresistance in the SQD9 model. **(A–C)** Where indicated, cells were treated with 10 nM of ETC complex III inhibitor antimycin A. **(A)** OCRs corresponding to basal respiration, maximal respiration and the oxidative contribution to ATP production (*n* = 7). **(B)** Cell numbers expressed relative to time 0 (*n* = 7–8). **(C)** Clonogenicity of cells irradiated at increasing doses of Ɣ-rays. The left graph shows surviving fractions and the right graph LD_50_ (*n* = 9). **(D)** ROS production (*n* = 3). **(E)** Clonogenicity of cells treated with increasing doses of H_2_O_2_ for 48 h. The left graph shows surviving fractions and the right graph EC_50_ (*n* = 9). ^∗∗∗^*P* > 0.005; **ns* P* > 0.05; using Student’s *t-*test (**A,D,E** *right*) or one-way ANOVA with Dunnett’s *post hoc* test (**C** *right*).

To test the hypothesis that increased OXPHOS causes radiosensitivity, we treated SQD9-res cells with antimycin A, a well-known inhibitor of complex III of the mitochondrial ETC (Trumpower and Katki, 1975). A dose of 10 nM was found to almost fully inhibit SQD9-res cell respiration (Figure 8A) with no cytotoxicity (Figure 8B). However, clonogenic assays showed that antimycin A did not sensitize the cells to irradiation (Figure 8C). We also tested rotenone and metformin, but the two ETC complex I inhibitors were cytotoxic at the doses necessary to inhibit cell respiration. Thus, increased OXPHOS was not responsible for acquired radioresistance.

High OXPHOS activities are often associated to higher mitochondrial ROS (mtROS) production (Muller et al., 2004), which could basally activate antioxidant defense systems (Ristow, 2014) and render cells more radioresistant. However, total ROS levels did not differ between SQD9-wt and SQD9-res cells (Figure 8D), and challenging the cells with increasing concentrations of H_2_O_2_ did not unravel a better resistance of SQD9-res compared to SQD9-wt cells (Figure 8E).

### Changes in Nuclear DNA Damage and Repair Do Not Account for Acquired Radioresistance in SQD9 Cancer Cells

Irradiation causes oxidative damage to DNA, SSBs and DSBs that are responsible for cell killing. To avoid death, cells can activate nuclear DNA repair, which involves a cascade of phosphorylations orchestrated by two serine/threonine kinases: ataxia telangiectasia and Rad3-related protein (ATR) for SSB repair and ataxia-telangiectasia mutated (ATM) for DSB repair (Borgmann et al., 2016). The two kinases are activated *via* phosphorylation by upstream sensors of DNA damage. Cell metabolism can modulate DNA repair by at least three mechanisms: chromatin remodeling, DSB repair and redox homeostasis (Turgeon et al., 2018). We therefore investigated whether oxidative SQD9-res were more efficient than glycolytic SQD9-wt cells to repair DNA.

**FIGURE 9:**
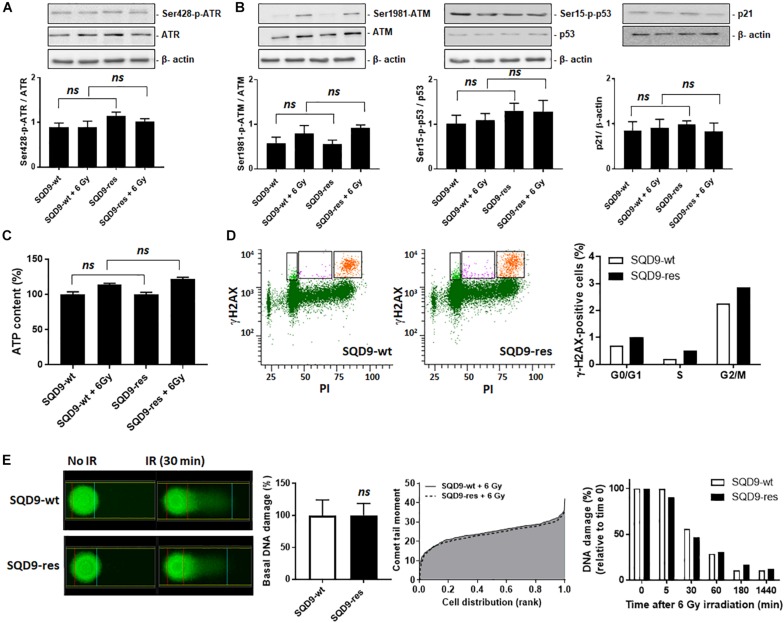
DNA damage repair is not different between radiosensitive and radioresistant SQD9 cells. **(A,B)** SQD9-wt and SQD9-res cells were analyzed 3 h after a 6 Gy γ-ray irradiation or sham treatment. **(A)** Representative western blots and graph showing Ser428-p-ATR/ATR expression (*n* = 8). **(B)** Representative western blots and graph showing Ser1981-p-ATM/ATM, Ser15-p-p53/p53 and p21/β-actin expression (*n* = 7–8). **(C)** ATP levels 2 h after a 6 Gy γ-ray irradiation or sham treatment (*n* = 8). **(D)** γ-H2AX cell content in relation to the cell cycle of cells irradiated with 8 Gy of γ-ray. In representative left graphs, γH2AX-positive cells are highlighted in orange. The right graph shows the position of γH2AX-positive cells in the cell cycle. Data are normalized for total cell number (*n* = 1–2). **(E)** DNA damage before and after a 6 Gy γ-ray irradiation. A representative Comet picture is shown on the far left, the middle left graph shows DNA damage before irradiation, the middle right graph shows the Comet tail moment (incorporating both % damaged DNA and DNA fragmentation) right after irradiation, and the far right graph shows DNA damage repair over time after irradiation (*n* = 2–3). *ns P* > 0.05; by Student’s *t*-test **(E)** or one-way ANOVA with Dunnett’s *post hoc* test **(A–C)**.

ATR activity (Ser428 phosphorylation, Figure 9A) and ATM activity (Ser1981 phosphorylation, Figure 9B *left*) did not differ between SQD9-wt and SQD9-res cells under basal conditions or 3 h after a 6 Gy irradiation. Downstream, ATM effectors phospho-Ser15-p53 and p21 (Kastan and Lim, 2000) behaved similarly in the two cell lines (Figure 9B *middle* and *right*). ATP levels were similar in SQD9-res compared to SQD9-wt cells at basal and 2 h after irradiation (Figure 9C). We further analyzed nuclear DNA damage using flow cytometry and histone H2AX phosphorylation (γH2AX) as a marker of DSBs. Ten minutes after a single 8 Gy irradiation, the percentage of γH2AX-positive cells in G2/M phase was similar for SQD9-wt and SQD9-res cells (Figure 9D). Furthermore, an alkaline Comet assay reporting on basal DNA damage, maximal irradiation-induced DNA damage (Comet tail moment) and the kinetics of DNA repair showed no difference when comparing SQD9-wt and SQD9-res cells (Figure 9E). We concluded that changes in DNA damage and repair did not account for the acquired resistance of SQD9-res cells.

### Compared to Radiosensitive, Radioresistant SQD9 Cells Have More Mitochondria

**FIGURE 10:**
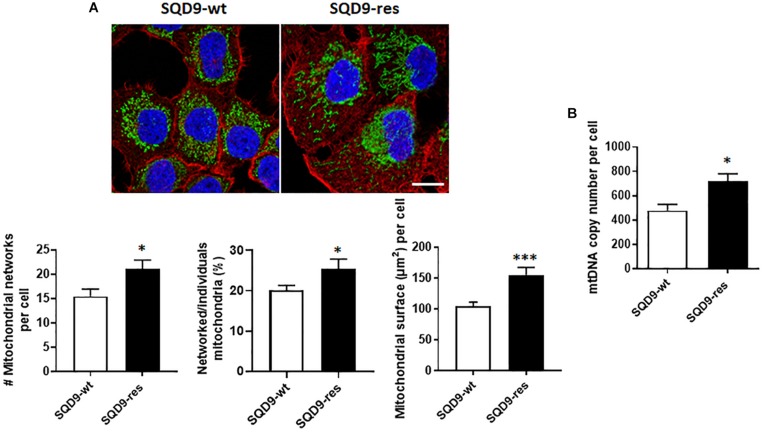
Mitochondrial abundance and morphology differ between radiosensitive and radioresistant SQD9 cells. **(A)** Mitochondria were labeled with an anti-Tom20 antibody (green), the cytoskeleton with an anti-actin antibody (red) and cell nuclei with DAPI (blue). Representative pictures are shown on top (bar = 20 μm), and graphs show the number of mitochondrial networks per cell (*left*), the ratio of networked/individual mitochondria (*middle*) and the relative surface of cells occupied by mitochondria (MitoFootprint, *right*) (*n* = 20–34). **(B)** mtDNA content normalized to nuclear DNA content (*n* = 8). ^∗^*P* < 0.05, ^∗∗∗^*P* < 0.005 by Student’s *t-*test.

We finally analyzed mitochondrial organization. Compared to SQD9-wt cells, mitochondrial labeling revealed that SQD9-res cells had a denser mitochondrial network occupying a larger volume per cell (Figure 10A). In particular, the networked/individual mitochondria ratio was higher in SQD9-res, indicating a better mitochondrial organization compared to SQD9-wt cells (Figure 10A *middle graph*). mtDNA content was ∼50% higher in SQD9-res compared to SQD9-wt cells (Figure 10B).

## Discussion

Our study aimed to identify whether metabolic differences related to mitochondrial metabolism could account for acquired radioresistance in human HNSCC cells. In order to differentiate intrinsic *versus* hypoxia-related radioresistance, all *in vitro* experiments were performed with unlimited oxygen supply. We first aimed to produce matched pairs of cells selected for radioresistance or for differential glucose uptake. Among all cellular models tested, SQD9 was the only one for which a selection for radioresistance and for differential glucose uptake was feasible, which might be due to the fact that the other cell lines were already radioresistant and/or metabolically homogeneous. We report that radioresistant SQD9 cells are more oxidative than radiosensitive SQD9 cells, and *vice versa*. Radioresistant cells also have a lower expression of HK1 and HK2. However, silencing HKs or inhibiting OXPHOS was not sufficient to suppress intrinsic radioresistance, which seemed to be primarily linked to mitochondrial abundance.

The major finding of our study is that, in SQD9 cells, acquired radioresistance is associated with increased OXPHOS and that cells selected for high OXPHOS are radioresistant. In both cases, increased OXPHOS was associated to a reduced expression of HK1 and HK2, with unchanged expression of GLUT1, GLUT3 and PKM2. Of note, if this change decreased the uptake and sequestration of glucose analog 2-NBDG in SQD9-res and SQD9-LGU cells, it did not reduce overall glucose consumption nor lactate production by SQD9-res cells, which can only be explained by an increased rate of glucose processing in the TCA cycle and in side pathways connected to glycolysis downstream of HKs. Accordingly, silencing *HK1*, *HK2* or both using siRNAs did not selectively radiosensitize SQD9-res and SQD9-LGU compared to SQD9-wt and SQD9-HGU cells, respectively. Another interesting observation was that both SQD9-wt and SQD9-res cells had no respiration spare capacity, whereas SQD9-LGU and SQD9-HGU cells had a respiration spare capacity. As such, this difference is not linked to the radiosensitivity of the cells. We rather believe that it is related to the heterogeneity of the cell populations, with SQD9-wt cells being more heterogeneous than the selected SQD9-res cells, themselves more heterogeneous than FACS-sorted SQD9-LGU and SQD9-HGU cells representing 2% of the initial SQD9-wt population. Whether metabolic exchanges between cell subpopulations account for more efficient anaplerosis in unsorted *versus* FACS-sorted cells remains to be determined, knowing that glucose and glutamine (provided by the medium) and lactate (produced by the cells) were not limiting in our experimental conditions.

That radioresistance and high OXPHOS are mutually associated established that mitochondria control the intrinsic radiosensitivity of SQD9 cells. At a first glance, this may seem in contradiction with the observations of Bansal et al. (2014) and Mims et al. (2015) who reported decreased glutamine-fueled respiration and increased glycolysis in radioresistant human SCC-61 HNSCC cells compared to wild types. Both SQD9 and SCC-61 cells are HPV-negative. However, while the SCC-61 cell selection protocol was similar to ours, the authors further cloned chronically irradiated cells on soft agar, which we believe could have introduced additional metabolic variables. One possibility is that their radioresistant SCC-61 clone represents cancer stem cells that are well known to resist to radiotherapy (Yamamoto et al., 2016) and generally have a distinct metabolic phenotype compared to a bulk heterogeneous population (Wong et al., 2017; Snyder et al., 2018). Accordingly, an increased dependency on aerobic glycolysis over OXPHOS has been reported in HNSCC cancer stem cells (Aguilar et al., 2016). Alternatively, several pathways accounting for radioresistance could have been enriched during SCC-61 cloning (Aguilar et al., 2016), and differences between the SQD9 and SCC-61 models could be inherent to the tissue of origin, the larynx and the tongue, respectively, and to a different genetic background.

As previously reported for both HPV-positive and HPV-negative HNSCC cancer cells (Cruz-Gregorio et al., 2019), the different types of metabolic adaptations affecting glycolysis, respiration, glutaminolysis, lipid metabolism and autophagy all converge to protect cells from irradiation by reducing redox stress and/or to accelerate cell repair by accelerating biosynthesis and energy production. We therefore logically expected that enhanced OXPHOS in SQD9-res cells would generate more ATP, more ROS basally activating redox defenses (mitohormesis) and would be a marker of superior mitochondrial integrity compared to SQD9-wt cells. However, this was not the case, as demonstrated by direct ATP and ROS measurements that did not strongly differ between the two cell variants, and there was no difference in cell survival following a H_2_O_2_ challenge. Intriguingly, the metabolic reprogramming that we identified did not protect nuclear DNA from irradiation damage, nor did it accelerate DNA repair. Indeed, we detected no difference in irradiation-induced nuclear DNA damage, no difference in the activation of ATR and ATM DNA repair pathways, and no difference in the rate of DNA repair. Yet, the mutual association between radioresistance and high OXPHOS and between high OXPHOS and radioresistance that we identified clearly pointed at mitochondria.

There was no difference in mtDNA genome integrity between radiosensitive and radioresistant cells. In particular, irradiation did not select for mitochondrial variants, and the two cell lines had similar *in vitro* and *in vivo* expansion rates. In fact, among all the possibilities that we tested that can directly influence radiosensitivity/radioresistance, only the number of mitochondria and mtDNA copy number singled out. Compared to SQD9-wt, SQD9-res cells had ∼50% more mitochondria that formed a denser network around the cell nucleus and ∼35% more mtDNA. We thus thought that mitochondria could have shielded the nucleus, with mtDNA acting as a bait to protect nuclear DNA from damage. However, this was not the case, as nuclear DNA damage measured right after irradiation was similar in SQD9-wt and SQD9-res cells. It remains that, compared to nuclear DNA, mtDNA is not condensed nor protected by histones and could therefore preferentially react with irradiation-generated ROS. In theory, a high number of mitochondria would offer an advantage against radiotherapy, in terms of preservation of mitochondrial functions and/or mitochondrial repopulation. Of course, this hypothesis should be tested experimentally in more than one cell line, with a special focus on mtDNA repair (Stein and Sia, 2017) and mitochondrial dynamics (fission and mitophagy, mitochondrial biogenesis and fusion) (Senft and Ronai, 2016). Our main hypothesis for future studies is that a high mitochondrial turnover repopulates radioresistant cancer cells faster with functional mitochondria, thus allowing them to better recover form irradiation, compared to radiosensitive cancer cells with a low mitochondrial turnover. Increased mitochondrial turnover would depend on increased fission and mitophagy to eliminate damaged mitochondria, together with increased mitochondrial biogenesis and fusion to repopulate cells with fit mitochondria. Interestingly, a high mitophagic rate has been reported to be associated with chemoresistance in several cancer cell types (Su et al., 2016; Villa et al., 2017; Yamashita et al., 2017; Naik et al., 2018), and Zheng et al. (2015) further observed that mitophagy participates in hypoxia-induced radioresistance in breast cancer cell lines. There is also good evidence that a high rate of mitochondrial biogenesis, primarily depending on activation of mitochondrial transcription factor A (TFAM) and transcription coactivator peroxisome proliferator-activated receptor γ coactivator-1α (PGC-1α), participates in chemoresistance (Gabrielson et al., 2014; Wu et al., 2016; Shen et al., 2018; Hu and Guo, 2019). TFAM and PGC-1α have not yet been linked to radioresistance, but Wang et al. (2017) recently showed that silencing mitochondrial single stranded DNA-binding protein (SSBP1), a protein that normally ensures mtDNA stability, decreased mtDNA copy number and sensitized H1299 human lung cancer cells to X-ray irradiation. If acquired radioresistance would rely on optimized mitochondrial dynamics, then it could potentially be reversed by targeting mitophagy (Panigrahi et al., 2019) and/or mitochondrial biogenesis (Wang et al., 2016) in cancer cells. Alternative hypotheses connecting mitochondria to radioresistance would involve differences in calcium transport and release and mitochondrial resistance to apoptosis. These possibilities warrant further investigation.

## Conclusion

In conclusion, we developed matched lines of human HNSCC SQD9 cells presenting high *versus* low radiosensitivity or high *versus* low oxidative metabolism. Using these models, we found that, among all metabolic changes, an increase in mitochondrial abundance is likely to participate in acquired radioresistance. Future studies should investigate mitochondrial dynamics in this model and in additional cancer cell lines in order to ultimately identify new radiosensitizing strategies possibly targeting mitochondrial turnover.

## Data Availability Statement

All datasets generated for this study are included in the article.

## Ethics Statement

All *in vivo* experiments were performed with approval of UCLouvain Comité d’Ethique pour l’Expérimentation Animale (approvals 2014/UCL/MD/014 and 2016/UCL/MD/018) according to national and European animal care regulations.

## Author Contributions

DG lead the experimental work, produced most data and analyzed all results. HM, LZ, VB, PD, and VG were involved in the production and analysis of metabolic data and clonogenic assays. MG, HS, AS, LD, and PL were involved in the production and analysis of mtDNA data. CB and DB were involved in the production and analysis of microscopy and FACS data respectively. MD was involved in the production and analysis of the Comet assay. VG, HS, AS, LD, PL, MD, and PS provided access to specific equipment and resources. PS directed the study, provided most resources, and analyzed all results. DG and PS wrote the manuscript. All authors critically read, edited, and endorsed the content of the manuscript.

## Conflict of Interest

PL was co-inventor of a patent on mtDNA (PCT/EP2014/059089). He has financial relationships with Oncoradiomics, ptTheragnostic/DNAmito, Health Innovation Ventures/BHV, ConvertPharmaceuticals and DualTpharma. Three softwares licensed to ptTheragnostic/DNAmito, Oncoradiomics and Health Innovation Ventures were used. The remaining authors declare that the research was conducted in the absence of any commercial or financial relationships that could be construed as a potential conflict of interest.
